# Top health service concerns: a data mining study of the Shanghai health hotline

**DOI:** 10.3389/fdgth.2025.1462167

**Published:** 2025-02-10

**Authors:** Lili Shi, Tong Zhao, Shimiao Shi, Tianyu Tan, Aksara Regmi, Yuyang Cai

**Affiliations:** ^1^ Xinhua Hospital, School of Medicine, Shanghai Jiao Tong University, Shanghai, China; ^2^ Shanghai General Hospital, Shanghai Jiao Tong University School of Medicine, Shanghai, China; ^3^ School of Public Health, Shanghai Jiao Tong University, Shanghai, China

**Keywords:** health hotline, text mining, concerns of health service, urban health governance, health public

## Abstract

**Objective:**

Our study aims to explore the health service issues of public concern through analyzing the basic characteristics of callers and information from the health hotline in Shanghai. The findings of this study will provide a reference to relevant government departments and assist the government in optimizing the allocation of health resources.

**Methods:**

Our research utilized 16,962 original work orders from the 12,320 health hotline, collected since 2015. We applied natural language processing (NLP) to analyze the content of these work orders, facilitating effective text mining and information extraction. Initially, we performed data cleaning to remove irrelevant information and protect caller privacy by anonymizing personal details. This cleaned data was then organized into a structured database for further analysis. Using text mining, we examined various aspects of the calls, including duration, purpose, and topics discussed, to identify patterns and themes that emerged.

**Results:**

The calls were categorized into four main groups: complaints, suggestions, inquiries, and requests for assistance. Complaints were the most frequent category, totaling 8,669 (51.11%), followed by help-seeking at 3,335 (19.66%), consultations at 2,727 (16.08%), and comments and suggestions at 1,484 (8.75%). The analysis revealed that men made 6,689 (56.88%), surpassing the 5,071 (43.12%) from women. Additionally, calls from parents numbered 2,126 (56.84%), slightly exceeding the 1,614 (43.16%) from children. The top 10 health service concerns identified in Shanghai included medical staff attitudes, medications, fees, registration, family planning, medical disputes, ambulance services, environmental health, illegal medical practices, and immunization.

**Conclusions:**

This study not only identifies critical issues within the Shanghai health service system but also offers actionable insights to inform targeted policy interventions. The high volume of complaints regarding service attitudes and medical expenses underscores the need for stronger policies to improve patient-provider communication and ensure transparency and fairness in healthcare costs. Additionally, the data reveals considerable public concern about the availability and quality of medical services, suggesting that existing policies on resource allocation and service delivery may not adequately meet population needs. The methodologies employed here can be applied to other urban health contexts, providing a valuable framework for improving public health strategies globally.

## 1 Background

The Shanghai 12,320 health hotline plays a crucial role in addressing the growing health needs of the general population. Its objectives include disseminating health prevention knowledge, public health policies, laws, and regulations. The hotline responds to a wide range of public health inquiries, raises awareness about disease prevention, and offers health counseling. Additionally, it guides residents in adopting scientific and health-oriented lifestyles while serving as a platform for receiving and addressing complaints and reports related to public health emergencies, providing medical guidance, and explaining medical test results (1).

The previous research on health hotlines has highlighted their potential in enhancing citizens' health literacy, conducting public health surveillance, and optimizing health resource allocation (2–6). Analyzing data from the Shanghai 12,320 health hotline can provide comprehensive understanding into the true health service needs of residents and the primary challenges facing the health sector in Shanghai. By addressing these issues, relevant government departments can implement targeted measures to optimize health resource distribution (7–9).

This research addresses a critical gap in public health service challenges in urban environments, particularly in rapidly growing cities like Shanghai. By identifying key concerns through advanced topic modeling techniques, this study not only illuminates immediate service issues but also presents a replicable model for other cities facing similar challenges. The findings have the potential to inform health policies that are more responsive to citizens' needs, ultimately contributing to improved health outcomes on a broader scale.

## 2 Methods

### 2.1 Data source

In this study, we analyzed 16,926 original work orders from the 2015 records of the Shanghai health hotline, serving as our primary research dataset. These work orders, documented by hotline personnel, contained information on voice calls from citizens reporting various issues. Each work order had a unique identifier and included details such as call time, locations mentioned, content records, processing descriptions, and responses to the caller. The staff of the Shanghai 12,320 health hotline meticulously reviewed the key calls received to ensure the accuracy and relevance of the feedback.

### 2.2 Data cleaning

Data cleaning is the prerequisite for data analysis. Initially, the text-based segments of the work orders underwent a desensitization process to protect the privacy of callers. We then handled missing values by removing seriously missing data such as “Document type.” Following this, we performed deduplication procedures to eliminate overlapping data entries, particularly those with redundant meanings, such as similarities between “response to citizens' key points” and “completion report” fields. This step ensured that only relevant and unique data points were retained for analysis.

### 2.3 Handling abnormal calls

Call Duration: Abnormal calls with unusually long or short durations were identified as outliers using statistical methods based on the Interquartile Range (IQR). Calls outside the range of Q1 − 1.5 × IQR and Q3 + 1.5 × IQR were flagged for review. Extreme values, such as calls lasting several hours or just a few seconds, were investigated and removed if found to be errors (e.g., technical issues or misrecorded times).

Call Frequency: Outliers in call frequency were identified by analyzing the number of calls per individual or group. Calls exceeding the 99th percentile or a predefined threshold were flagged as abnormal. After review, we excluded errors or anomalies, but retained valid calls representing rare events, such as a caller with multiple health concerns.

Manual Review: A manual review of flagged calls was conducted to distinguish between legitimate cases (emergencies) and data errors (system glitches or duplicates). Invalid calls were excluded, while valid outliers were retained for analysis.

By combining statistical methods with manual review, we ensured that the dataset remained clean, reliable, and accurately represented health service concerns.

### 2.4 Fields included in the analysis

**Figure 1:**
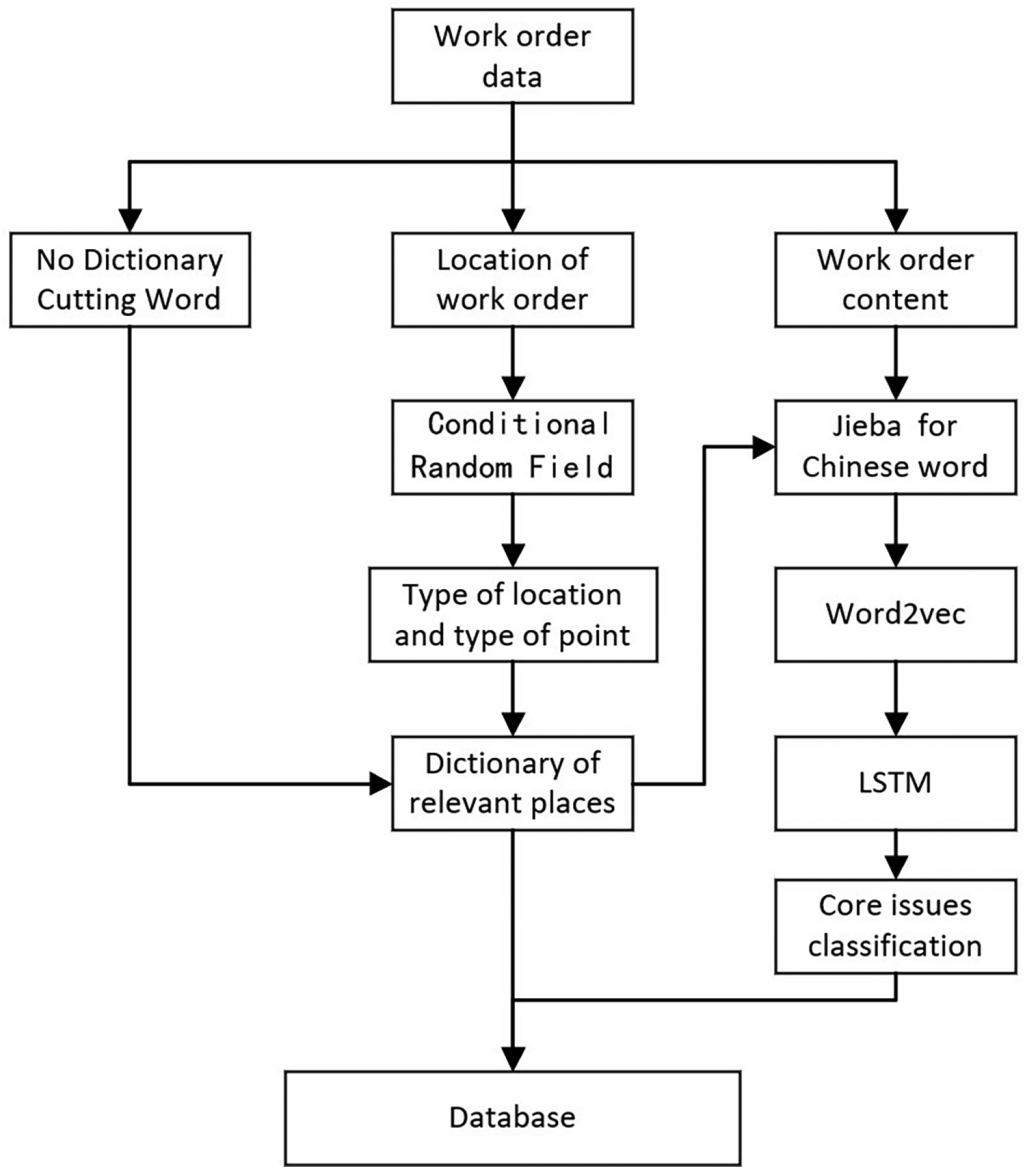
Text mining process.

After the data cleaning process and thorough discussions among research team and 12,320 technical experts, we have excluded fields that lack correlation, and fields with stronger correlation are retained as follows:

(1) Call Time: This records the precise moment when each work order was generated, allowing us to analyze the distribution of incoming calls over 24 h and across the 12 months of the year.
(2) Caller Gender: This indicates the gender of the individual making the call.
(3) Caller Object: This captures the identity of the caller, such as husband, wife, or parent, acknowledging that callers may report issues related to family members rather than themselves.
(4) Type: The purposes and contents of the calls were categorized into five groups: Complaint, Help-seeking, Consultation, Suggestion, and Others.
(5) Descriptive Information: This field provides context-specific descriptions of the calls, including details of reported problems, the purpose of the calls, public appeals, and requests for responses from the call center. The text mining process is illustrated in Figure 1.

### 2.5 Natural language processing

In this study, we applied a “No Dictionary Cutting Word” method for segmenting Chinese text, a language without explicit word delimiters. The technology employs statistical models, machine learning, or deep learning to infer word boundaries from context and usage patterns while dynamically adapting to new terms, making it particularly effective for languages with compound word structures. Combined with expert knowledge, this approach was essential for tasks such as keyword extraction and topic modeling, as shown in Figure 1.

We analyzed the “Descriptive Information” field, which includes detailed reports from residents on health service issues, using Latent Dirichlet Allocation (LDA). LDA is an effective algorithm for discovering latent topics in large text corpora, like those from the Shanghai health hotline (10, 11). Its probabilistic nature models each work order as a mixture of topics, revealing diverse concerns of the callers. LDA groups words based on co-occurrence, organizing documents into distinct topics despite variations in terminology.

We optimized LDA parameters to improve model performance and interpretability, focusing on:

Number of Topics (k): We conducted a grid search over k values from 5 to 50 in increments of 5 and identified 30 as optimal, balancing model complexity and data fit, with a coherence score of 0.55.
Hyperparameters (*α* and *β*): Testing α values between 0.01 and 0.1 revealed *α* = 0.05 as best for capturing multiple topics per document. For *β*, values between 0.01 and 0.1 suggested *β* = 0.02 produced the most coherent topics.

We validated the LDA model through a two-step process: manual topic labeling and comparison with the model's output, iteratively refining it for better interpretability. This dual approach ensured both statistical rigor and practical relevance.

For example, topics labeled as “service attitude” consistently included words like “communication,” “neglect,” and “impatience,” demonstrating high thematic coherence.

The LSTM model was further evaluated with parameter tuning for learning rates and batch sizes, achieving an F1-score of 0.87, precision of 0.85, and recall of 0.89. Comparisons with Random Forest (F1 = 0.81) and CNN (F1 = 0.83) demonstrated LSTM's superior ability to capture sequential dependencies in text. This robustness, along with optimal parameter settings, justified LSTM's selection for our classification tasks.

The insights from LDA, combined with the LSTM model's performance, provide a comprehensive understanding of public health service concerns (12). This framework can be applied to other urban health systems, broadening the scope of the findings and contributing to discussions on health policy. Additionally, analyzing the geographical distribution of complaints and comparing them with international studies enriched our analysis, offering evidence for informed policy recommendations (10).

## 3 Result

### 3.1 Classification of work orders

A comprehensive analysis was conducted on a total of 16,692 work orders from 2015, categorized into five primary types: complaints, help-seeking, consultations, suggestions, and others. Complaints made up the majority, representing 8,669 (51.11%) of the work orders, while help-seeking constituted 3,335 (19.66%), as detailed in Table 1.

**Table 1:** Classification and quantity of call records of Shanghai health hotline in 2015.

Classification	Quantity	Percent (%)
Complaints	8,669	51.11
Help-seeking	3,335	19.66
Consulting	2,727	16.08
Opinion suggestion	1,484	8.75
Other	162	4.40
Total	16,962	100.0

To provide a clearer understanding of these classifications, below are specific examples of work orders from each category:

Consultation: The caller did not receive the one-time family planning and only child fee. They have now heard that they may be eligible for the 5,000 yuan only child fee. They hope the management department can verify this and inform them where they can go to collect it.
Suggestions: A citizen called to report that there is widespread medicine waste. For example, a doctor prescribed three boxes of medication, but the citizen only needed two boxes to recover. The remaining medication was wasted, but there is no place to return it. Some people end up selling the leftover medication to scalpers. The citizen suggests that the management department establish medication return points, both paid and unpaid, to collect unused medicines. This would help make better use of the medication and prevent waste.
Complaint: On June 18, 2015, while receiving saline infusion on the second floor of the emergency department, there was a patient who caused a disturbance during the process. The caller reported the issue to the three medical staff responsible for the infusion, hoping they would advise the patient. However, the staff showed a cold attitude and did not address the concern.

Subsequent analysis of the main categories revealed the top five sub-categories for complaints, help-seeking, consultations, and suggestions, as summarized in Table 2.

**Table 2:** Sub-categories and quantity of Shanghai health hotline call logs in 2015.

Category	Sub-categories	Quantity
Help-seeking	Medical institutions and services	2,879
	Population and family planning	166
	Public health agencies and services	94
	Public health	91
	Medical reform policy	53
Complaints	Medical institutions and services	7,968
	Medical institutions and services	281
	Public health	245
	Population and family planning	79
	About 12,320	36
Suggestions	Medical institutions and services	1,224
	Population and family planning	67
	Public health	60
	Public health agencies and services	57
	Medical reform policy	53
Consultation	Seeking medical advice	944
	Population and family planning	875
	Medical institutions and services	176
	Policies and regulations	139
	Medical reform policy	101

### 3.2 Call time statistics

**Figure 2:**
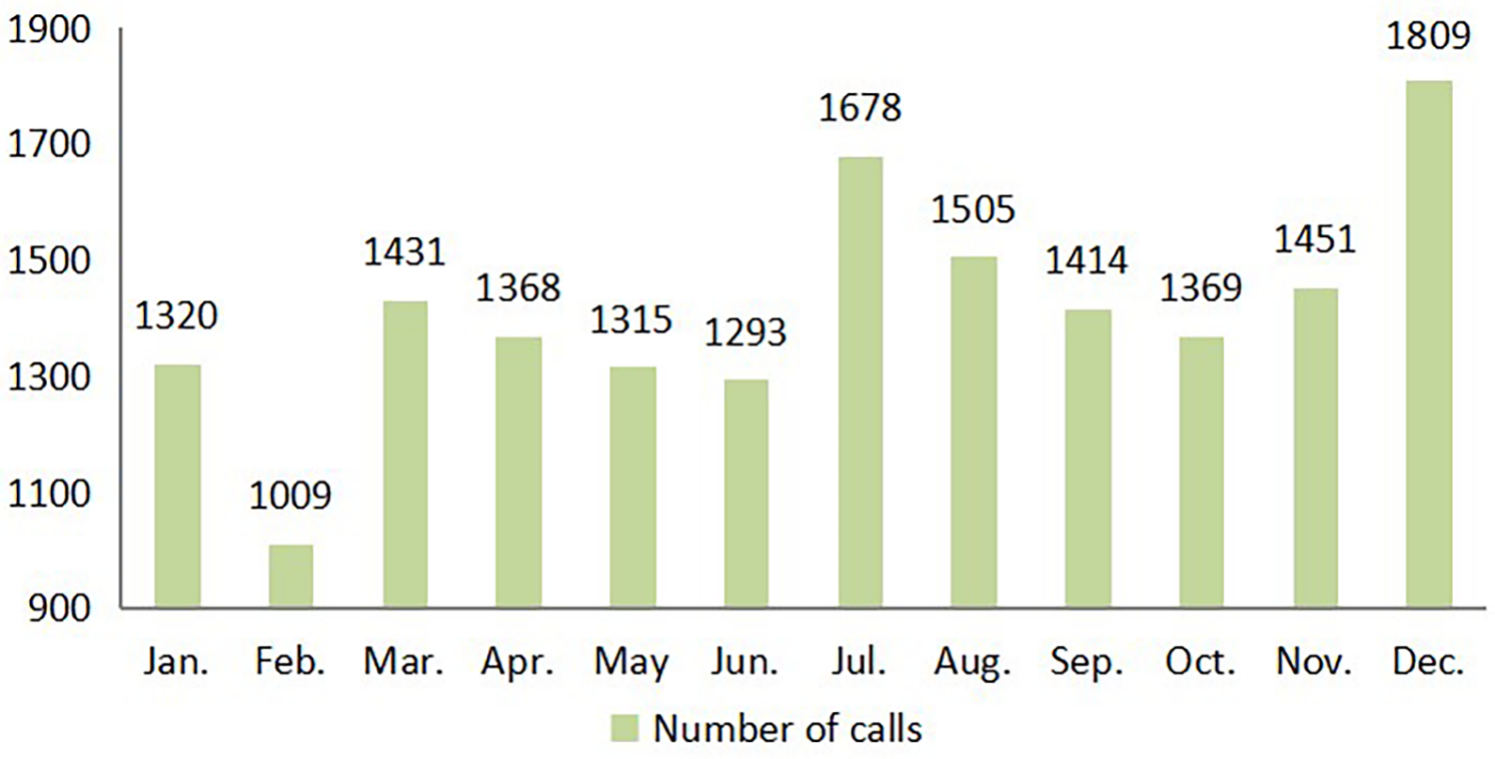
Monthly statistics of call logs in 2015.

From a yearly perspective, December recorded the highest volume of calls, exceeding 1,800, while February had the lowest call volume. Throughout the year, a fluctuating upward trend was observed, as illustrated in Figure 2.

**Figure 3:**
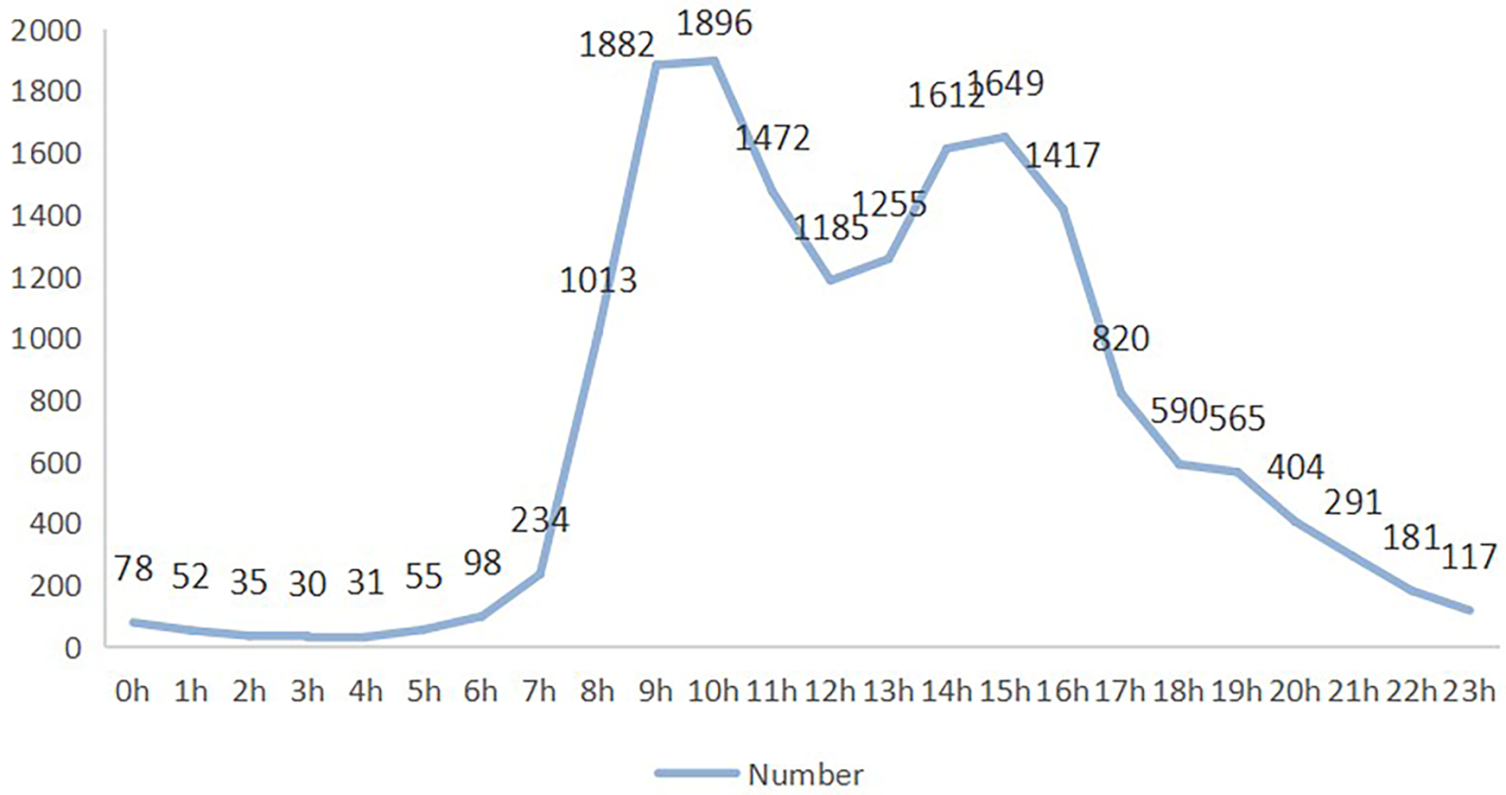
Period statistics of call records in 2015.

On a daily basis, the peak number of calls occurred between 10 AM and 11 AM, with busy periods from 9 AM to 12 PM and 2 PM to 4 PM. Calls during nighttime were relatively low, as shown in Figure 3.

### 3.3 Analysis of callers

The data indicates that there were 6,762 male callers and 5,118 female callers, showing a higher overall number of male callers. In Table 3, the statistical significance of gender differences within each call classification category is explicitly indicated. Gender differences were significant in the categories of complaints, consulting, opinion suggestions, and praise (*p* < 0.01), denoted by “***”. On the other hand, in the help-seeking category, the gender difference is not statistically significant, marked as “ns” (not significant).

**Table 3:** Gender differences in call classifications.

Classification	Male (Number, %)	Female (Number, %)	Significance
Complaint	3,519 (52.6%)	2,385 (47.0%)	***
Consulting	1,103 (16.5%)	1,126 (22.2%)	***
Help-seeking	1,178 (17.6%)	866 (17.1%)	ns
Opinion suggestion	756 (11.3%)	472 (9.3%)	***
Others	133 (2.0%)	222 (4.4%)	***

*** Indicates a statistically significant result with *p* < 0.001, showing a highly significant relationship between the variables presented in the table.

**Figure 4:**
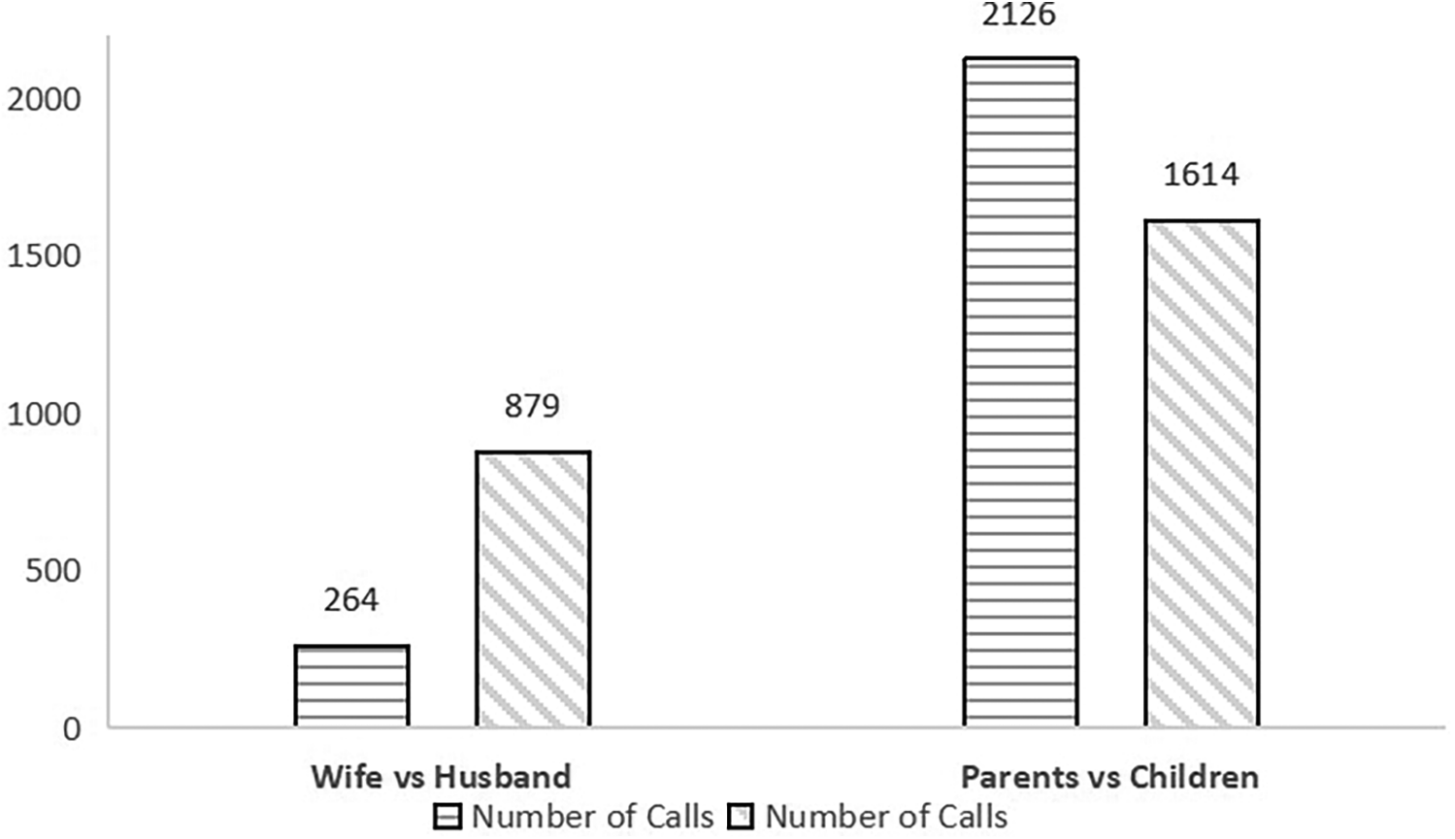
Caller objects of Shanghai 12,320 health hotline in 2015.

The results of the incoming caller analysis, as illustrated in Figure 4, reveal significant disparities in call patterns. Parents contributed a total of 2,126 calls, while children accounted for 1,614 calls. In contrast, the number of calls made by wives, at 264, was notably lower than those by husbands, who registered 879 calls. Importantly, the combined sum of calls from parents and children surpassed that of wives and husbands.

### 3.4 Correlation analysis of key health concerns

To explore the systemic connections among critical health concerns, Pearson and Spearman correlation analyses were conducted. The results are summarized in Table 4, highlighting significant relationships that underscore underlying systemic issues within the healthcare framework. In conducting the correlation analysis, we focused on the most pertinent relationships that directly address the core objectives of our study. These correlations collectively demonstrate the intricate interplay between service quality, administrative efficiency, financial factors, and patient behaviors.

**Table 4:** Pearson and spearman correlation coefficients among key health concerns.

Variable A	Variable B	Pearson r	Pearson *p*-value	Spearman *p*	Spearman *p*-value
Service Attitude	Registration Process	0.55	<0.01	0.53	<0.01
Service Attitude	Medical Disputes	0.65	<0.01	0.63	<0.01
Registration Process	Illegal Medical Practices	0.3	<0.05	0.28	<0.05
Medical Expenses	Illegal Medical Practices	0.4	<0.05	0.38	<0.05
Doctor-Patient Communication	Medical Expenses	0.58	<0.01	0.55	<0.01

### 3.5 The top ten health service issues concerned by public

**Figure 5:**
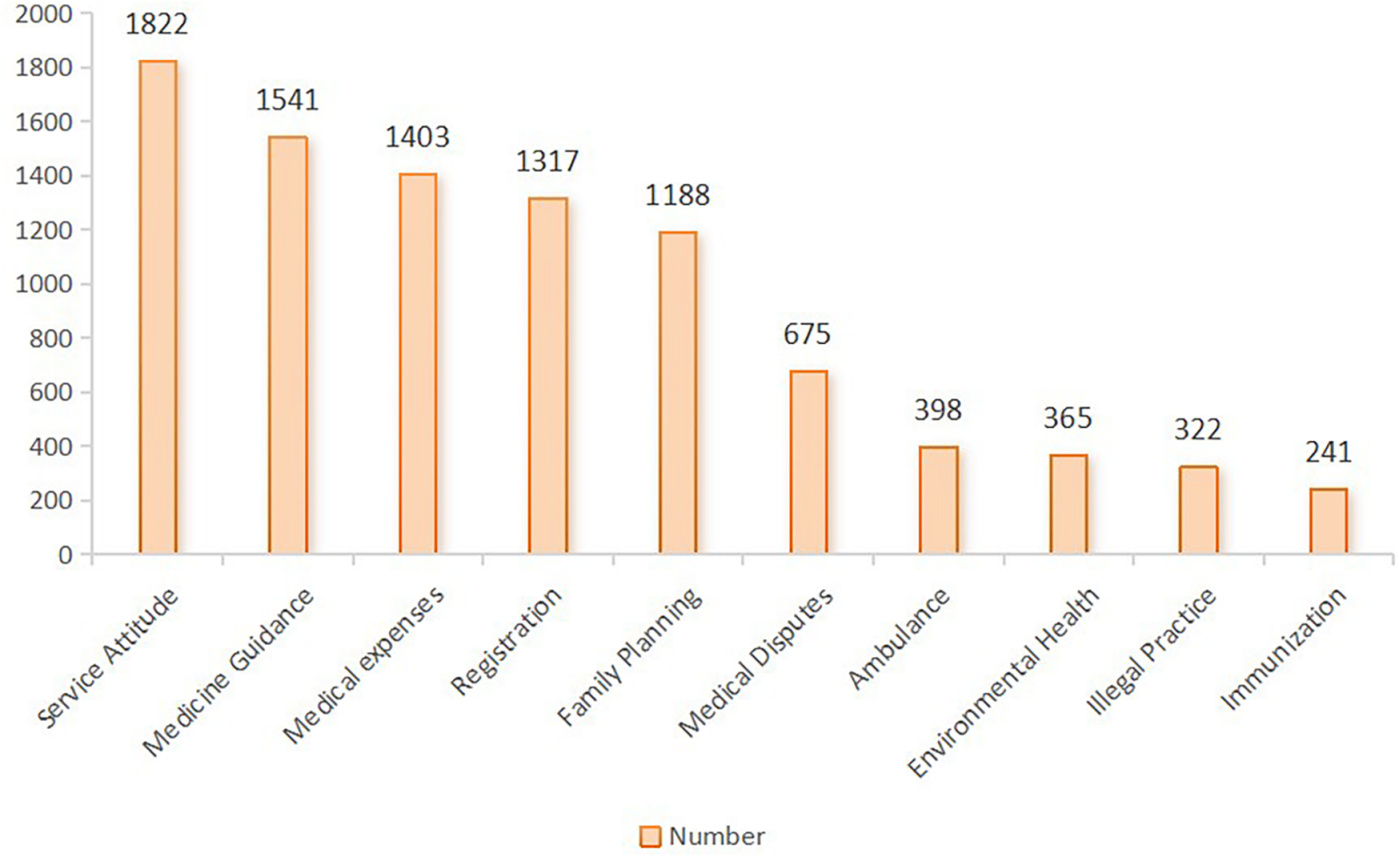
Top ten health service issues concerned by mostly public.

The findings presented in Figure 5 highlight the top ten health service issues raised by citizens, which include service attitude, medication guidance, medical expenses, registration, family planning, medical disputes, ambulances, environmental health, illegal practice of medicine, and immunization. In 2015, issues related to medical institutions and services accounted for a significant 77.44% of all work orders, with citizens primarily concerned about service quality, medical inquiries, fees, and registration. Additionally, there were concerns related public health issues, including environmental health and vaccination. On the other hand, certain subjects, like family planning-related issues, arose from policy shifts, exemplified by the change from the one-child policy to the two-child policy.

#### 3.5.1 Service attitude

In the 2015 analysis of health service-related concerns reported by citizens through the Shanghai health hotline, the predominant issue identified pertained to the service staff members' attitudes, with a mere 12.8% of feedback expressing positive experiences. This data underscores a significant disparity between patient expectations and the actual service delivered by healthcare professionals. Complaints categorized under service attitude can be divided into two main subtopics: “poor attitude” and “negligence of work.”

The subcategory “poor attitude” summarizes situations of insufficient communication and interpersonal interaction between healthcare providers (doctors and nurses) and patients, occasionally mentioning administrative staff. The report frequently describes specific behaviors that lead to negative patient experiences, including:

Reluctance in Communication: Patients recounted experiences where healthcare providers exhibited a marked unwillingness to engage in meaningful dialogue, crucial for understanding treatment plans and addressing health concerns. Dismissal of Patient Concerns: A concerning number of reports were really disturbing, indicating how medical staff were ignoring or even belittling patient concerns and worries, making them feel marginalized in the process of being taken care of. Standoffish Demeanor: Some healthcare workers maintained a distant or cold demeanor, creating an unwelcoming environment. Poor Explanation: A reluctance to provide detailed explanations left patients confused about their health conditions and treatment options, often leading to anxiety and frustration. Impatience: Instances of impatience during consultations were noted, with some doctors failing to communicate with patients throughout their visits, undermining the patient's understanding of their health.

The second subtopic, “negligence of work,” further shows that occasionally the medical staff were absent or unavailable during times when their services were needed. Limited Availability: Patients found it hard to access doctors for follow-up consultations or in case of emergency complications, and they were often attended to when conditions became too severe. Distraction Among Staff: Accounts that were put forward also observed medical personnel to be distracted, engrossing themselves in personal activities like talking over phones. Adding further woes is the fact that even medical staff has been reported to be engaged in verbal misconduct against patients. This includes scolding, yelling, and use of inappropriate language against the patients.

#### 3.5.2 Medication guidance

An analysis of 1,541 calls revealed four primary areas of concern: medical guidance (43.5%), drug purchases (33.4%), medication prescriptions (17.2%), and adverse drug reactions (5.9%).

Medical Guidance: There is a strong demand for assistance in navigating healthcare services, with many callers seeking recommendations for departments or facilities that specialize in specific conditions. This indicates a critical gap in accessible, patient-centered navigation support. Additionally, concerns about service availability during holidays highlight a mismatch between healthcare access and patient needs, potentially leading to delayed care.

Drug Access Issues: Many citizens face challenges obtaining specific medications, particularly those that are only available at select hospitals or pharmacies. Reports of discontinued or hard-to-find drugs reflect broader issues in pharmaceutical supply chains and underscore the need for policies to ensure drug availability, particularly for under-served populations.

Prescription Process Barriers: Issues related to drug prescriptions, such as the need for re-registration due to drug shortages, add unnecessary burdens on patients and signal systemic inefficiencies.

Adverse Drug Reactions: Reports of adverse reactions, including allergies and symptoms like fever and vomiting during vaccination or medication, further complicate issues of patient care and safety. These findings, therefore, call for systemic improvement to enhance access and efficiency in healthcare.

#### 3.5.3 Medical expenses

An in-depth analysis of financial grievances within the healthcare system reveals systemic issues tied to billing practices and financial operations. These concerns are grouped into several categories: “unreasonable fees,” “extra charges,” “refund problems,” and “medical insurance payment issues,” each of which highlights unique challenges embedded within the healthcare landscape.

Unreasonable Fees: This category includes instances where costs for examinations, treatments, or medications are significantly higher at certain facilities compared to others or previous pricing for similar services. This raises concerns about pricing transparency and consistency across institutions. Extra Charges: The issue revolves around patients being billed for services or items they did not receive or use, or instances where the billed quantities surpass actual usage. This highlights notable gaps in billing accuracy and overall integrity.

Refund Problems: The obstacles faced by patients when attempting to secure reimbursements after discontinuing medical services. Procedural inefficiencies, bureaucratic obstacles, and excessive documentation requirements often lead to extended processing times, all of which negatively impact patient satisfaction and perceptions of financial fairness.

Medical insurance payments: Less common but still critical, particularly when patients are prescribed non-covered medications without prior notification—raise crucial issues surrounding informed consent and communication during medical treatment.

Collectively, these financial grievances underscore the urgent need for systemic reforms aimed at enhancing transparency, accuracy, and fairness within healthcare financial operations.

#### 3.5.4 Registration

The challenges within the healthcare registration process underscore systemic inefficiencies that hinder access and service delivery. Key issues include difficult registration procedures, the “scalper phenomenon,” shortcomings in appointment services, and repeated registration, all of which significantly impact patient care and public health.

The issue of difficult registration highlights significant barriers to accessing healthcare services, driven by factors such as: complex registration procedures, limited availability of specialized healthcare professionals, and a high demand for certain medical services. Such complexities not only frustrate patients but also prevent timely access to necessary medical care, exacerbating delays in treatment.

The “scalper phenomenon,” as it relates to healthcare, involves individuals—known as hospital scalpers—who secure appointment slots or registration tickets for healthcare services, later reselling them at inflated prices. This practice particularly preys on patients in urgent need of medical attention, exploiting systemic inefficiencies, such as lengthy wait times and flawed appointment scheduling systems, which often limit access to medical professionals or specialized treatments.

Complaints related to appointment registration services further expose the technological and procedural deficiencies within the current system. Problems such as unsuccessful phone appointments, invalid online registrations, insufficient online quotas, and extended waiting times reflect a substantial mismatch between the healthcare system's capacity and the needs of a population of over 25 million citizens.

Lastly, the concept of “repeated registration” describes the frustration patients face when required to register again, often incurring additional costs, if their examination cannot be conducted on the same day as their initial registration. In many cases, this results in appointments being delayed beyond 24 h, compounding the financial burden on patients and exposing significant inefficiencies in hospital scheduling practices.

#### 3.5.5 Family planning

A striking 47.6% of inquiries centered on the family planning incentive policy, indicating strong public is keen to understand the financial and logistical supports for families under the new framework. These incentives, such as financial subsidies and extended maternity and parental leave, underscore the socio-economic challenges families face when considering a second child.

Additionally, citizens raised concerns about newborn care and the capacity of existing healthcare systems to support growing family sizes, addressing vital topics like healthcare services, vaccination schedules, and childcare availability. This indicate a broader societal concern about the readiness of existing healthcare and social support systems to accommodate the policy shift.

#### 3.5.6 Medical disputes

In an analysis of hospital department-specific complaints, surgery, internal medicine, and obstetrics-gynecology emerged as primary focal points, accounting for 28.7%, 25.7%, and 16.2% of total disputes, respectively. These figures not only pinpoint departments most prone to patient dissatisfaction but also shed light on the varied nature of these complaints. In parallel, disputes within surgical departments frequently involved cases of misdiagnosis or improper surgical procedures, underscoring the need for more accurate diagnostic practices and improved surgical precision. Within internal medicine, grievances often revolved around misdiagnoses and the perceived overuse of treatments and medications. In obstetrics-gynecology, common complaints concerned misdiagnosis and issues related to pregnancy and childbirth management. Across these departments, a shared issue was the lack of a patient-centered approach, with breakdowns in communication between medical staff and patients contributing to misunderstandings and dissatisfaction. Allegations of over-treatment and unnecessary prescriptions further pointed to a disconnect between patient care objectives and actual medical practices. Additionally, specific complaints regarding forced specialist slot allocation and errors in vaccine administration highlighted operational inefficiencies and lapses in clinical safety, emphasizing the need for systemic improvements.

#### 3.5.7 Ambulance

The analysis of keywords such as “ambulance” and “120” (the emergency hotline number) highlights four critical issues within emergency medical services: prolonged wait times following emergency calls, hesitancy to respond to non-emergent situations, a shortage of available ambulances, and instances where pre-booked ambulance services were canceled or reassigned to other tasks. The issue of delayed ambulance response times after calling 120 reflects a significant breakdown in response efficiency, potentially worsening patient outcomes. Meanwhile, the reluctance to respond to non-emergency calls underscores a delicate challenge within the emergency service framework, highlighting the need to balance limited resources between emergency and non-emergency situations. The limited availability of ambulances signals deeper resource inadequacies within the 120 system. Furthermore, the practice of canceling or reallocating pre-booked ambulance services raises serious concerns regarding the reliability and predictability of medical transport. This not only diminishes public trust in the 120 system but also highlights the urgent need for improved operational guidelines to safeguard the integrity of pre-booked services. Addressing these issues is essential for enhancing the overall effectiveness and reliability of emergency medical response systems.

#### 3.5.8 Environmental health

The data reveals a wide range of public concerns regarding environmental hygiene, with key issues including public smoking, water quality, noise pollution, and indoor air quality. Public smoking emerged as the primary concern, comprising approximately 39.7% of the total calls. Complaints primarily originated from public spaces such as malls, supermarkets, and restrooms, highlighting the health risks associated with involuntary exposure to second-hand smoke.

Water quality issues constituted 30.4% of environmental health-related calls, with specific grievances concerning the clarity and safety of swimming pool water and the efficacy of water filtration systems. These concerns highlight the importance of establishing and enforcing rigorous water quality standards, coupled with regular monitoring and maintenance protocols for public and recreational water systems to prevent waterborne diseases and ensure public safety. Noise pollution, primarily associated with hospital renovations, the operation of large machinery (e.g., air-conditioners), and logistical activities involving medical equipment (e.g., oxygen tanks), underscores the need for effective noise management practices in healthcare settings. Indoor air pollution, especially formaldehyde contamination in newly decorated gyms and healthcare facilities, poses significant health risks, including respiratory issues and allergic reactions. Citizen complaints concerning formaldehyde encompass several aspects, including reports of excessive formaldehyde levels in their immediate environments and inquiries regarding the regulatory standards for formaldehyde concentrations. Additionally, there are queries about where formaldehyde testing services can be obtained. A myriad of additional environmental health concerns was also identified, including but not limited to, hotel hygiene, inadequate lighting, mosquito infestations, restroom cleanliness, and radiation exposure. Addressing these issues is vital for enhancing public health and safety within communities.

#### 3.5.9 Illegal medical practice

The operation of illegal clinics within the healthcare system reveals significant weaknesses in public health governance and regulation. These unauthorized facilities, often lacking proper equipment and qualified personnel, present serious health risks. Notably, illegal dental, cosmetic, and abortion clinics are among the most common offenders, highlighting specific areas of healthcare vulnerable to such illicit activities. This trend reflects broader systemic issues related to healthcare access and regulatory enforcement.

The rise of these illegal clinics can be attributed to several factors. Firstly, the high demand for dental, cosmetic, and abortion services, combined with inadequacies or high costs in the formal healthcare system, drives individuals to seek alternatives. Secondly, the relatively low technical and facility requirements for operating these clinics further enable unqualified practitioners to establish illegal operations, significantly endangering patient safety and the quality of care.

Furthermore, the operation of clinics without proper medical qualifications not only jeopardizes individual health outcomes but also undermines the integrity of the entire healthcare system. Patients treated by unqualified practitioners face the risks of misdiagnosis, inappropriate treatment, complications, and potential life-threatening dangers. Moreover, the lack of regulatory compliance in these environments, particularly concerning disinfection and hygiene practices, can lead to the spread of infectious diseases, exacerbating public health issues.

The uneven distribution of medical resources also plays a key role in the proliferation of illegal clinics. Disparities in access, affordability, and quality of healthcare across different regions create gaps that these unauthorized practices exploit. This not only poses a direct threat to public health but also highlights the critical need for healthcare system reforms to close these gaps and ensure equitable access to quality medical care.

#### 3.5.10 Immunization

The analysis outlines two main categories of communication: inquiries about vaccination services and suggestions for improvements. The survey predominantly revolves around the logistics of immunization services, indicating a strong public interest in accessing vaccinations. Specific queries highlight the need for clear and understandable information about institutions providing vaccinations, locations, and operating hours.

Moreover, inquiries related to allergies reflect public concerns about vaccine safety, with citizens seeking information on potential allergic reactions and side effects post-immunization. The most frequently mentioned vaccines in these surveys are Hepatitis B, Influenza, and Chickenpox, suggesting a higher awareness and demand for these specific vaccines among the public. Suggestions received through hotlines underscore constructive public opinions on expanding and optimizing immunization services. Proposals such as setting up additional vaccination sites and extending vaccination hours indicate a desire among citizens to improve the accessibility of vaccination services. These suggestions also imply a recognition that current operational limitations may pose barriers to achieving higher vaccination rates.

## 4 Discussion

This discussion comprehensively examines the importance of evaluating healthcare-seeking behaviors, complaints, suggestions, and inquiries within the healthcare system, as they reflect public satisfaction and pinpoint areas requiring improvement. This holistic understanding of residents' interactions provides a nuanced perspective on healthcare, identifying strengths and areas for reform.

Findings from the Shanghai health hotline underscore critical aspects of residents' healthcare needs, particularly difficulties in accessing medical care and high costs. The lack of a structured general practitioner (GP) and referral system in China often leads patients to bypass primary care and directly seek treatment at well-known tertiary hospitals, regardless of the severity of their conditions (13, 14). The influx of patients with minor ailments and chronic diseases into these top-tier hospitals reduces system efficiency and compromises the quality of care for all (15, 16).

This situation highlights a key systemic challenge: healthcare providers, overwhelmed by the volume of patients, often lack the capacity to deliver personalized or attentive care, leading to dissatisfaction and complaints. Notably, dissatisfaction with the attitudes of healthcare workers is a leading cause of complaints, accounting for 26.9% of total grievances, while excessive waiting times represent 21.7% (17, 18). A moderate positive correlation was identified between service attitude and the registration process. This suggests that a negative service attitude may either contribute to or result from a cumbersome registration process. Inefficient registration procedures can lead to patient frustration, thereby diminishing their overall perception of service quality. These service shortcomings reflect the strain of occupational burnout among healthcare workers, which, in turn, creates a feedback loop. Poor service attitudes reduce patient satisfaction, further increasing stress on medical staff and diminishing the quality of care—a cycle that exacerbates public discontent with the healthcare system (17). Moreover, a strong positive correlation was observed between service attitude and medical disputes. This indicates that poorer service attitudes among medical service providers are significantly associated with an increase in medical disputes.

A significant positive correlation exists between the registration process and illegal medical practice. Difficulties encountered during the registration process may compel patients to bypass official channels and seek unauthorized medical services. Scalpers exploit inefficiencies in the hospital registration system by occupying appointment slots and reselling them at inflated prices, further aggravating inequitable access to healthcare services. This practice imposes significant financial burdens on patients. The analysis revealed that, A moderate positive correlation was found between medical expenses and illegal medical practices. High medical costs may incentivize patients to circumvent the formal medical system in favor of more affordable yet illegal alternative care options, where safety and quality cannot be assured. These challenges not only increase health risks but also perpetuate systemic inefficiencies, as unresolved cases eventually return to the formal healthcare system. The lack of an effective registration system, combined with overcrowding, has further eroded public trust in the healthcare system. Many patients suspect underlying systemic issues such as favoritism or corruption, which continue to strain the relationship between the healthcare system and its users (17).

Although the number of complaints related to illegal medical practice decreased in 2018 compared to 2014, the underlying cause lies in the continued demand for low-cost medical services (19). Addressing these interconnected challenges requires targeted reforms. These include establishing a robust GP and referral framework to manage patient flow, improving registration systems to eliminate scalper activities, and implementing measures to reduce healthcare worker burnout. Such reforms are critical to alleviating hospital overcrowding, improving equity and efficiency, and restoring public confidence in the healthcare system.

The inefficiencies highlighted in the appointment process call for a reassessment of existing technological infrastructure. Implementing digital billing and online registration systems offers a viable solution to improve operational efficiency. These systems enhance transparency, reduce administrative burdens for patients and providers, and address problems associated with scalpers and duplicate registrations, ensuring fairer access to medical services (20).

In addition to service attitudes, medical quality, and doctor-patient relationships, costs are a significant source of public dissatisfaction (21–25). From 2010 to 2016, government subsidies accounted for only 7.71%–9.13% of total public hospital revenue (26–31), underscoring their heavy reliance on operational income. This low level of government support exacerbates the tendency for physicians to act as “economic agents,” focusing on maximizing personal or institutional revenue (31, 32). The phenomenon of “provider-induced demand,” where healthcare providers influence patients to consume unnecessary services, is a notable concern. This is driven by information asymmetry, as providers possess more medical knowledge and may not always prioritize the patient's best interests when recommending services (33). A significant correlation was detected between doctor-patient communication and medical expenses. Consequently, patients may misinterpret medical costs as excessive or unreasonable, particularly when they lack a clear understanding of treatment plans, diagnostic necessity, or cost breakdowns. Such perceptions, compounded by unmet expectations during treatment, further fuel dissatisfaction and concerns over overtreatment (34–41).

Research indicates that although residents' incomes experienced significant growth in 2015, the annual growth rate of medical expenses (9.95%) surpassed the income growth rates for urban (8.72%) and rural residents (8.44%). This disparity places a disproportionately heavier financial burden on lower-income rural residents. Consistent with the high proportion of complaints related to medical expenses observed in this study's hotline data, this trend underscores the escalating concerns over medical costs, which are intensifying both the demand for and dissatisfaction with healthcare services among residents (42).

Shanghai has implemented various policies to address these issues, including capping the number of patients doctors can see daily at 40 and setting a minimum consultation time of 10 min per patient (43). To strengthen the GP and referral system, tertiary hospitals must reserve 30% of specialist appointments for referrals from family doctors (44). Additionally, the government has introduced measures to regulate medical fees and consultation procedures while promoting “extended prescriptions,” allowing patients to obtain medications without frequent hospital visits, improving overall satisfaction.

Despite these efforts, there remains room for targeted policy improvements. Enhancing communication between patients and providers through empathy-focused training programs could significantly improve the patient experience. Standardizing pricing models within healthcare institutions would increase transparency, while strengthened regulatory oversight could ensure patients are informed of treatment costs upfront, fostering greater trust in the system.

Further efforts should focus on improving healthcare access and expanding capacity in high-demand areas. Increasing the number of healthcare professionals, extending clinic hours, and optimizing appointment scheduling systems would reduce wait times and enhance access to medical services. Additional policies could include improving healthcare oversight, combating scalper activities, and providing subsidies for rare disease medications and essential drugs to incentivize continued production. This initiative to improve access to healthcare services is closely related to changes in social structures, particularly when policy changes and seasonal diseases are involved, as healthcare demand fluctuates accordingly.

The introduction of China's two-child policy on October 29, 2015. According to statistics from the Shanghai Municipal Health Commission, the proportion of second-child births among Shanghai's registered population reached 27.42% in 2016, highlighting the significant impact of the “two-child policy” on family planning decisions (45). Combined with this study's hotline data, a noticeable increase in calls regarding family planning policy consultations was observed in 2015, indicating a rapid rise in public demand for related services following the policy's implementation. Especially during the winter months, the combined effects of seasonal diseases and policy changes have driven a surge in hotline calls, highlighting how these factors jointly shape healthcare demand. Daily call peaks often align with work hours, indicating that residents are more likely to seek medical assistance during daytime working hours. This pattern reveals the relationship between social activity and healthcare demand.

The high demand for healthcare services among men results from a combination of societal and cultural expectations, personal health awareness, health risks, and social roles. Men are more likely to seek emergency or specialized medical services in urgent or chronic care situations rather than preventive or primary healthcare services. This tendency is influenced by how men perceive and respond to health issues, shaped by social and cultural norms (46). Additionally, men are often driven to access healthcare services more frequently due to work-related injuries or health concerns. This behavior is also associated with differences in health awareness and behaviors, which are closely tied to gender roles and societal expectations (47, 48).

These gender differences in health behaviors are also closely linked to family roles and the involvement of parents in healthcare services, especially in managing children's health. Parents' socioeconomic status, family roles, and responsiveness to their children's health needs collectively drive their frequent use of healthcare services. Parents, particularly mothers, play a central role in managing their children's health needs and navigating the healthcare system (49). This responsibility within the family structure makes parents more proactive in utilizing health services. Studies have highlighted that children's health needs, combined with the socioeconomic status of their parents, contribute significantly to parents' higher frequency of accessing healthcare services (50).

Finally, because the continuous monitoring and evaluation mechanisms are essential, in future studies, health hotline data could be analyzed post-policy implementation to assess the effectiveness of these measures. The Shanghai health hotline itself can be a valuable tool for ongoing feedback, allowing policymakers to gauge the effectiveness of implemented policies and make necessary adjustments. Health hotline data could be analyzed post-policy implementation to assess the effectiveness of these measures. Furthermore, the application of LDA in this context demonstrates its utility in public health research, providing a method for uncovering latent issues that may not be immediately apparent through traditional survey methods.

By addressing the root causes of dissatisfaction and enhancing the efficiency of the healthcare system, these reforms can contribute to better health outcomes for the population. Additionally, these findings and recommendations could serve as a valuable model for other urban centers in China that are grappling with similar healthcare challenges, showcasing the importance of data-driven policy-making in urban health governance.

## 5 Conclusion

The analysis of call data to the Shanghai 12,320 health hotline reveals important trends in public health service concerns. December saw the highest call volume, while February had the lowest, indicating seasonal fluctuations in health service demands. Additionally, demographic patterns, such as notably higher male participation and significant parental concerns, highlight intergenerational health service needs. This observation aligns with previous studies (51).

Citizens' feedback and complaints can provide important feedback for health services and serve as valuable tools for improving the quality of health services (52–57). The analysis reveals significant public dissatisfaction with current medical services, particularly highlighting prevalent complaints about service attitudes, medication guidance, and medical costs. These findings underscore the urgent need to strengthen medical infrastructure and optimize resource allocation to effectively address the public's pressing healthcare issues.

The insights gained from this study are crucial for enhancing urban health governance and shaping health policy. Advocating for healthcare reforms that focus on improving patient-provider interactions, providing clear medication instructions, and revising billing and insurance practices to reduce patient financial burdens, we can significantly enhance the quality of health services. By implementing data-driven policies and making service improvements, we can boost public trust in the healthcare system, ultimately contributing to the overall well-being of urban residents.

## 6 Limitations

The primary data source for this study was the Shanghai 12,320 health hotline, serving a city renowned for its high medical standards that exceed those in many other parts of China. While Shanghai's vast geography and large population make this data valuable for understanding urban healthcare needs and improving services, it may not fully represent the experiences of all residents across the country. Individuals who choose not to use the hotline or those living in rural or less developed regions may have different health concerns and behaviors. The urban-centric nature of the data likely introduces certain biases into the study. For example, the higher prevalence of complaints about medical expenses and service attitudes may reflect the expectations and demands of an urban population that is more accustomed to higher service standards and has greater financial means.

Issues such as access to healthcare services, which may be a significant concern in rural areas, are less likely to be captured by the hotline data from Shanghai. Consequently, the study may understate the severity or nature of healthcare challenges in less developed regions, where access to basic health services, affordability, and the availability of skilled healthcare professionals are more pressing issues. Future research should consider incorporating data from rural and less developed regions to provide a more comprehensive understanding of health service needs across China. Expanding the scope of data sources would enhance the generalizability of the findings and help address the diverse healthcare challenges present in different regions. Additionally, triangulating hotline data with other sources, such as national health surveys or data from rural health centers, would enhance the validity of the findings. This cross-validation can ensure a more balanced representation of the population's health concerns and capture the nuances that may be overlooked when relying solely on hotline data.

By addressing these limitations and incorporating more diverse data sources, future studies can contribute to a more nuanced and comprehensive understanding of healthcare challenges across China, ultimately leading to more effective and inclusive health policies.

The study primarily relies on self-reported data from hotline calls, which is prone to biases including recall and social desirability biases. These biases could skew the accuracy of the data regarding healthcare experiences and perceptions.

Meanwhile, natural language processing still faces challenges in Chinese. Like a constructed language, a complete, finite set of rules cannot define the vast Chinese vocabulary and diverse sentences. There is also much ambiguity. Additionally, language processing often involves massive knowledge bases that are costly to build and maintain.

## Data Availability

The original contributions presented in the study are included in the article/Supplementary Material, further inquiries can be directed to the corresponding authors.
